# Stable biomarker discovery in multi-omics data via canonical correlation analysis

**DOI:** 10.1371/journal.pone.0309921

**Published:** 2024-09-09

**Authors:** Taneli Pusa, Juho Rousu

**Affiliations:** Department of Computer Science, Aalto University, Espoo, Finland; Institute of Apicultural Research, CHINA

## Abstract

Multi-omics analysis offers a promising avenue to a better understanding of complex biological phenomena. In particular, untangling the pathophysiology of multifactorial health conditions such as the inflammatory bowel disease (IBD) could benefit from simultaneous consideration of several omics levels. However, taking full advantage of multi-omics data requires the adoption of suitable new tools. Multi-view learning, a machine learning technique that natively joins together heterogeneous data, is a natural source for such methods. Here we present a new approach to variable selection in unsupervised multi-view learning by applying stability selection to canonical correlation analysis (CCA). We apply our method, StabilityCCA, to simulated and real multi-omics data, and demonstrate its ability to find relevant variables and improve the stability of variable selection. In a case study on an IBD microbiome data set, we link together metagenomics and metabolomics, revealing a connection between their joint structure and the disease, and identifying potential biomarkers. Our results showcase the usefulness of multi-view learning in multi-omics analysis and demonstrate StabilityCCA as a powerful tool for biomarker discovery.

## Introduction

Multi-omics analyses have emerged as the next generation of high-throughput methods. By simultaneously considering multiple levels of the biological system under study, multi-omics offers a more comprehensive view of it, and a better chance of understanding the underlying processes [1–3].

However, along with promise come challenges: issues typical of biological data sets such as small sample sizes combined with a large number of variables are further compounded in multi-omics data [4]. Heterogeneous data types and large discrepancies in the number of variables between different omics are also inherent problems [5, 6]. Taking full advantage of multi-omics necessitates developing and adopting new analytical methods [7, 8].

The large number of variables encountered in biological data necessitates feature selection, and the primary goal of omics studies is often precisely the identification of a core set of variables of interest: biomarker discovery. This can be achieved via regularisation of models and imposing sparsity. However, picking the correct level of sparsity is a non-trivial task. In addition, there exists a known trade-off between sparsity and stability: how sensitive is the choice of variables to changes in the data [9, 10].

Ideally, multi-omics analyses will go beyond simply concatenating different data types, preserving their complementary nature. Multi-view learning, the umbrella term for machine learning methods that consider the integration of multiple feature sets, is well-suited to accomplish this task [3, 11].

One potential solution to the sparsity-stability trade-off is offered by the stability selection framework [12, 13]: by simulating data perturbation with a subsampling procedure, more stable feature sets can be found. Here we show how stability selection can be efficiently applied in a multi-view context.

Our approach, named StabilityCCA, combines canonical correlation analysis (CCA) with stability selection to find subsets of connected variables in a multi-omics data set. CCA is an unsupervised multi-view method that finds a pair of maximally correlated projections for two sets of variables [14]. By applying the stability selection framework to CCA, we offer an alternative approach to variable selection in high-dimensional multi-view analysis. Instead of setting a fixed level of model sparsity by optimising regularisation parameters, StabilityCCA constructs a multitude of models with varying levels of sparsity, giving a more holistic view of variable importance.

We tested StabilityCCA with simulated data and two different types of multi-omics from an inflammatory bowel disease (IBD) gut microbiome data set. The results show improvements in variable selection performance, in particular selection stability. Importantly, we saw significant improvements in stability in real data. We further demonstrate the method by analysing the canonical correlations found in the IBD data, uncovering a connection between the metagenome-metabolome structure and IBD status. With StabilityCCA, we were able to identify the important variables behind this connection, many of which are known IBD biomarkers.

This article is organised as follows. In a theory section, we introduce CCA, sparse CCA and stability selection, and show how stability selection can be applied to sparse CCA, leading to StabilityCCA. In a results section, we show results for two types of simulated data and a real multi-omics data set, comparing two different sparse CCA methods to their StabilityCCA extensions. The article ends with a discussion of the results.

## Methods

### Canonical correlation analysis

Canonical correlation analysis is an unsupervised dimensionality reduction method that can be applied when the variables under study naturally group into two disjoint sets. Suppose we have two corresponding sets of observations $X\in R^{n\times p_{x}}$ and $Y\in R^{n\times p_{y}}$ where *n* stands for the number of observations, and *p*_*x*_ and *p*_*y*_ for the number of variables in **X** and **Y** views respectively. We assume that the columns of **X** and **Y** have been standardised to have mean zero. CCA seeks projections **X** **a** and **Y** **b** such that the correlation between them is maximised:

$$\begin{array}{c} \underset{a,b}{\text{max}}\frac{\left\langle Xa,Yb\right\rangle }{{∥Xa∥}_{2}{∥Yb∥}_{2}} \end{array}$$ (1)

where 〈⋅, ⋅〉 denotes the dot product.

The solution to Eq (1) $a\in R^{p_{x}}$ and $b\in R^{p_{y}}$ are the first pair of *canonical coefficients*, and the projections $u=Xa\in R^{n}$ and $v=Yb\in R^{n}$ the first pair of *canonical variables* or scores. Further pairs can then be found by requiring that they are uncorrelated with the previous ones, up to min({rank(**X**), rank(**Y**)}) pairs.

The CCA problem can be solved analytically, for example using the singular value decomposition [15]. However, when the number of variables exceeds that of observations, this solution is no longer unique. A classic remedy is to introduce some form of regularisation, typically inducing sparsity in the coefficient vectors **a** and **b**, and maximise the CCA objective via optimisation [16–19], resulting in *sparse CCA* (SCCA).

Rather than adding a penalty term to the objective, as is commonly done in regularised regression methods, many SCCA approaches instead formulate the regularisation into a set of constraints on the coefficient vectors **a** and **b**. An optimal solution can then be found via alternating optimisation in which the objective is iteratively improved from one side at a time, imposing the regularisation constraints at each step. Such an approach has been previously employed by [18], in a closely related formulation by [20], and in the context of kernel CCA by [19].

We will adopt and test two different SCCA approaches to be used as the base procedure with StabilityCCA. The first one, due to Witten *et al.*, is based on a penalised matrix decomposition, and makes the simplifying assumption that the covariance matrices **X**^*T*^**X** and **Y**^*T*^**Y** can be treated as diagonal, effectively maximising the numerator in Eq (1). A combination of *L*_1_- and *L*_2_-norm constraints is used to induce sparsity, giving the following objective:

$$\begin{array}{cccc} & \underset{a,b}{\text{max}} & & \left\langle Xa,Yb\right\rangle \end{array}$$ (2)

$$\begin{array}{cccc} & \text{s.t.} & & {∥a∥}_{2}^{2}\leq 1,{∥b∥}_{2}^{2}\leq 1 \end{array}$$ (3)

$$\begin{array}{cccc} & & & {∥a∥}_{1}\leq c_{x},{∥b∥}_{1}\leq c_{y} \end{array}$$ (4)

where *c*_*x*_ and *c*_*y*_ are regularisation parameters for **a** and **b** respectively. This can be optimised by an alternating algorithm where at each step one of the coefficient vectors is kept fixed and Eq 2 maximised which can be done exactly (for full details of the algorithm, see [18]). We will refer to this method as penalised matrix decomposition CCA (PMD-CCA).

PMD-CCA has several attractive qualities for our purposes. Firstly, when $1<c_{x}<\sqrt{p_{x}}$, the *L*_1_ and *L*_2_ constraints interact, setting some coefficients in **a** to zero, giving us the feature that the coefficient vector will be maximally sparse at *c*_*x*_ ≤ 1, and no sparsity is imposed at $c_{x}\geq \sqrt{p_{x}}$. This will prove very convenient for StabilityCCA. Secondly, the optimisation algorithm has low time complexity and appears to converge fast when a good initialisation is given (as proposed by [18], first singular vectors of the cross-covariance matrix appear to be a good choice in practice). This is important since the stability selection framework will necessarily add some further computational burden.

Our second SCCA method is essentially a combination of the approach used by [19] and PMD-CCA. That is, we retain the *L*_1_-*L*_2_ constraints but solve for the full CCA objective:

$$\begin{array}{cccc} & \underset{a,b}{\text{max}} & & \frac{\left\langle Xa,Yb\right\rangle }{{∥Xa∥}_{2}{∥Yb∥}_{2}} \end{array}$$ (5)

$$\begin{array}{cccc} & \text{s.t.} & & {∥a∥}_{2}^{2}\leq 1,{∥b∥}_{2}^{2}\leq 1 \end{array}$$ (6)

$$\begin{array}{cccc} & & & {∥a∥}_{1}\leq c_{x},{∥b∥}_{1}\leq c_{y} \end{array}$$ (7)

Optimisation is done using an alternating projected gradient algorithm (for details, see [19]). We will refer to this method as SCCA-EC for “SCCA with Elastic-net Constraints”.

The SCCA-EC method has the same convenient qualities regarding the regularisation and low time complexity. However, contrary to PMD-CCA it does not ignore the within view covariances. It is not exactly clear when this assumption is appropriate and so it is interesting to see if and when it makes a difference. Furthermore, previous results suggest that different SCCA methods might be better suited for different types of data [21], so it makes sense to try different approaches with the StabilityCCA framework.

### Stability selection

One potential solution to the sparsity-stability trade-off is offered by the stability selection framework, described in detail in [12, 13]. The idea is to simulate data perturbation by repeatedly subsampling the data. For each subsample, a regularisation path is calculated, forming a sequence of models with varying levels of sparsity. By recording the frequency with which a variable was selected at a given level of sparsity, we can estimate the probability of each variable to be selected as a function of regularisation.

Here we show how to apply stability selection to CCA. As introduced in the previous Section, we will test two different SCCA approaches as base procedures but the framework can in principle be applied with any SCCA method.

Since we have two sparsity parameters, we sample the parameter space using pairs of parameter values (*c*_*x*__*i*_, *c*_*y*__*i*_). To capture the full range of regularisation, we run the parameters from (1, 1) to $\left(\sqrt{p_{x}},\sqrt{p_{y}}\right)$, populating the interval using a logarithmic sequence. This will sample the lower end of the parameter space more densely, effectively giving it more weight, since we are more interested in relatively sparse models where only a few variables are being selected.

**Fig 1:**
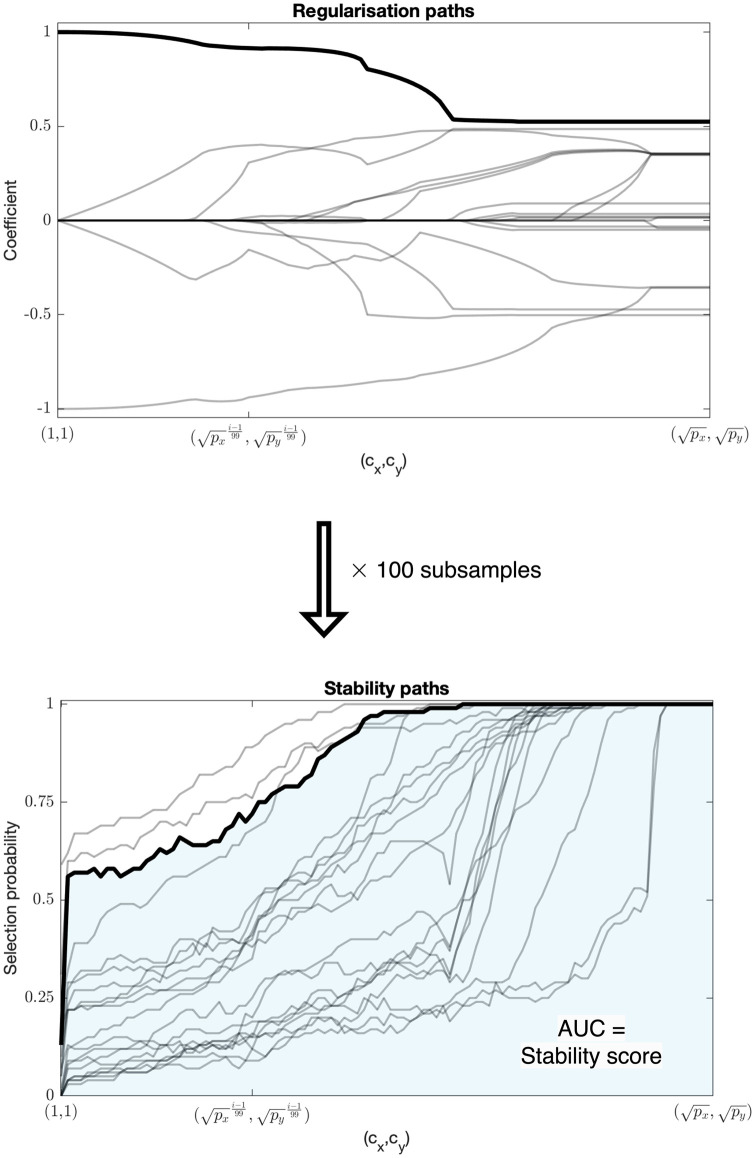
Schematic representation of the StabilityCCA procedure. Above are shown regularisation paths: the SCCA model as a function of sparsity. Below, stability paths, derived from the regularisation paths for 100 subsamples of size *n*/2. The stability score of a variable is the area-under-curve (AUC) of its stability path.

The procedure, *StabilityCCA*, is described in pseudocode in Algorithm 1 and illustrated schematically in Fig 1 which shows a visual representation of the output: a *stability path*. It shows for each variable its selection probability, as estimated by StabilityCCA, as a function of the sparsity parameters.

We define the *stability score* of a variable as the area-under-curve of its stability path. In other words, it is the average selection probability across all (*c*_*x*_, *c*_*y*_) values sampled. It can be seen as a measure of both relative and absolute variable importance: variables with higher stability scores are selected more often and thus more likely to be true signal variables. Values close to one indicate that a variable is selected almost always, even in very sparse models, and is likely to be very important for the model.

We tested two variable selection strategies based on the stability score, both using a single hyperparameter. In “Top-k” selection, variables are ranked and the top-k variables are selected, *k* being the hyperparamater. In threshold selection (Thr), variables with a stability score above a threshold *τ* will be selected, and *τ* is the hyperparameter. With both strategies, a CCA model can then be formed by running the base procedure with only the selected variables and regularisation parameters set so that no further sparsity is induced.

**Algorithm 1 StabilityCCA**

**Input: X**, **Y**: Data matrices

**Output:** P_*X*_, P_*Y*_: Selection probabilities

 *S* ← set of subsamples

 *C* ← sequence of parameter value pairs

 **for** (*X*_*s*_, *Y*_*s*_) ∈ *S* **do**

  **for** (*c*_*x*__*i*_, *c*_*y*__*i*_) ∈ *C* **do**

   (**a**, **b**) ← SCCA(*X*_*s*_, *Y*_*s*_, *c*_*x*__*i*_, *c*_*y*__*i*_)

   count_*X*__*ij*_ ← count_*X*__*ij*_ + **1**(**a**_*j*_ ≠ 0)

   count_*Y*__*ij*_ ← count_*Y*__*ij*_ + **1**(**b**_*j*_ ≠ 0)

  **end for**

 **end for**

 P_*X*__*ij*_ = count_*X*__*ij*_/|*S*|

 P_*Y*__*ij*_ = count_*Y*__*ij*_/|*S*|

⊳ *S* is a set of 100 subsamples derived by randomly splitting *X* and *Y* in half 50 times.

⊳ *C* is defined as ${\left({\sqrt{p_{x}}}^{\frac{i-1}{99}},{\sqrt{p_{y}}}^{\frac{i-1}{99}}\right)}_{i=1}^{100}$.

⊳ SCCA is SCCA-EC or PMD-CCA.

## Results

### Simulated data

#### Data

We generated simulated data with a known ground truth using two different data models. The first we will refer to as “single latent data”. Let **a**^true^ and **b**^true^ be ground truth coefficient vectors for the first (**X**) and second (**Y**) view respectively, and $p_{x}^{\text{true}}$ and $p_{y}^{\text{true}}$ the number of true variables. We randomly select $p_{x}^{\text{true}}$ indices *A*^true^ in **a**^true^ to be the true variables (same for **b**^true^). The entries $A_{i}^{\text{true}}$ are then drawn from the uniform distribution $U_{\left[0,1\right]}$ if *j* ∈ **a**^true^, and set to zero otherwise (same for **b**^true^). A latent factor $Z_{i}∼N\left(0,1\right)$ is drawn for each sample *i* and the data is generated with $X_{ij}∼N\left(Z_{i}a_{j}^{\text{true}},0.5\right)$ and $Y_{ij}∼N\left(Z_{i}b_{j}^{\text{true}},0.5\right)$.

In the second data model, “multivariate data”, **a**^true^ and **b**^true^ are generated in the same manner. Data is then drawn from a multivariate Gaussian distribution N(0,Σ) where

$$\begin{array}{ccccc} \Sigma =[\begin{array}{cc} \Sigma_{XX} & \Sigma_{XY}\\ \Sigma_{YX} & \Sigma_{YY} \end{array}], & & {\Sigma_{XX}}_{ij}=\{\begin{array}{cc} 1, & i=j\\ 0.5, & i\neq j;i,j\in A^{\text{true}}\\ 0, & \text{otherwise} \end{array}, & & \Sigma_{XY}=a^{\text{true}}{b^{\text{true}}}^{T} \end{array}$$ (8)

and equivalently for **Σ**_*YY*_ and **Σ**_*YX*_.

Let now *s*_*x*_ be the true sparsity of the **X**-view, that is, $s_{x}=p_{x}^{\text{true}}/p_{x}$ (and equivalently for **Y**). For all simulations, we set *s*_*x*_ = 2*s*_*y*_, $p_{x}^{\text{true}}=10$ and $p_{y}^{\text{true}}=15$, and run simulations for *s*_*x*_ values 1/2, 1/4 and 1/10. We set *n* = 50 in all cases. This set-up is meant to capture to varying degrees of severity many of the potential challenges encountered in multi-omics data: the curse of dimensionality, and asymmetry in the number of true variables, the level of true sparsity and number of variables between the two views.

#### Metrics

We evaluated the methods’ ability to identify true variables using two metrics. The area under the receiver operating characteristic curve (AUC) was calculated using the absolute values of canonical coefficients as the score for SCCA-EC and PMD-CCA, and the stability scores for their StabilityCCA equivalents. Balanced accuracy (BA) was calculated for binarised canonical coefficients or for the selections performed with the Top-k and the Thr procedure for StabilityCCA.

The stability of selected variable sets was evaluated using the stability estimator introduced by [22]. The stability estimator is a value between 1 and $-\frac{1}{M-1}$ where *M* is the number of variable sets. The maximum value 1 is achieved only when all selected sets are exactly equal and the minimum when their intersection is empty. The expected value of a random selection where each subset is equally likely to be drawn is zero.

Finally, model performance was evaluated by applying the canonical coefficients to a test set of size 100 and calculating the resulting correlation. For each scenario, we generated ten different random ground truths **a**^true^,**b**^true^, and ten different training and test set pairs for each **a**^true^,**b**^true^. Results are reported as averages over these 100 training/test sets for AUC, BA and test set correlation. The stability estimator was calculated over each of the sets of ten training sets with the same ground truth and we report the average over these.

#### Hyperparameter tuning

All hyperparameter tuning was done within the training set using ten rounds of 3-fold cross-validation and test set canonical correlation as metric of success. For the base procedures alone, SCCA-EC and PMD-CCA, a grid search over evenly distributed values of *c*_*x*_ and *c*_*y*_ in the range $\left[1,\sqrt{p_{x}}\right]$ and $\left[1,\sqrt{p_{y}}\right]$ was performed with grid size 15. For the Thr selection procedure, 100 values evenly distributed between the highest and lowest stability score were tested. For Top-k selection, since the number of potential *k* values can be very large, a binary search strategy was employed between 1 and *p*_*x*_ + *p*_*y*_. Note that in practice, one would likely aim for low values of *k*. However, we chose this agnostic tuning strategy so as to not artificially bias the model towards sparse models.

**Fig 2:**
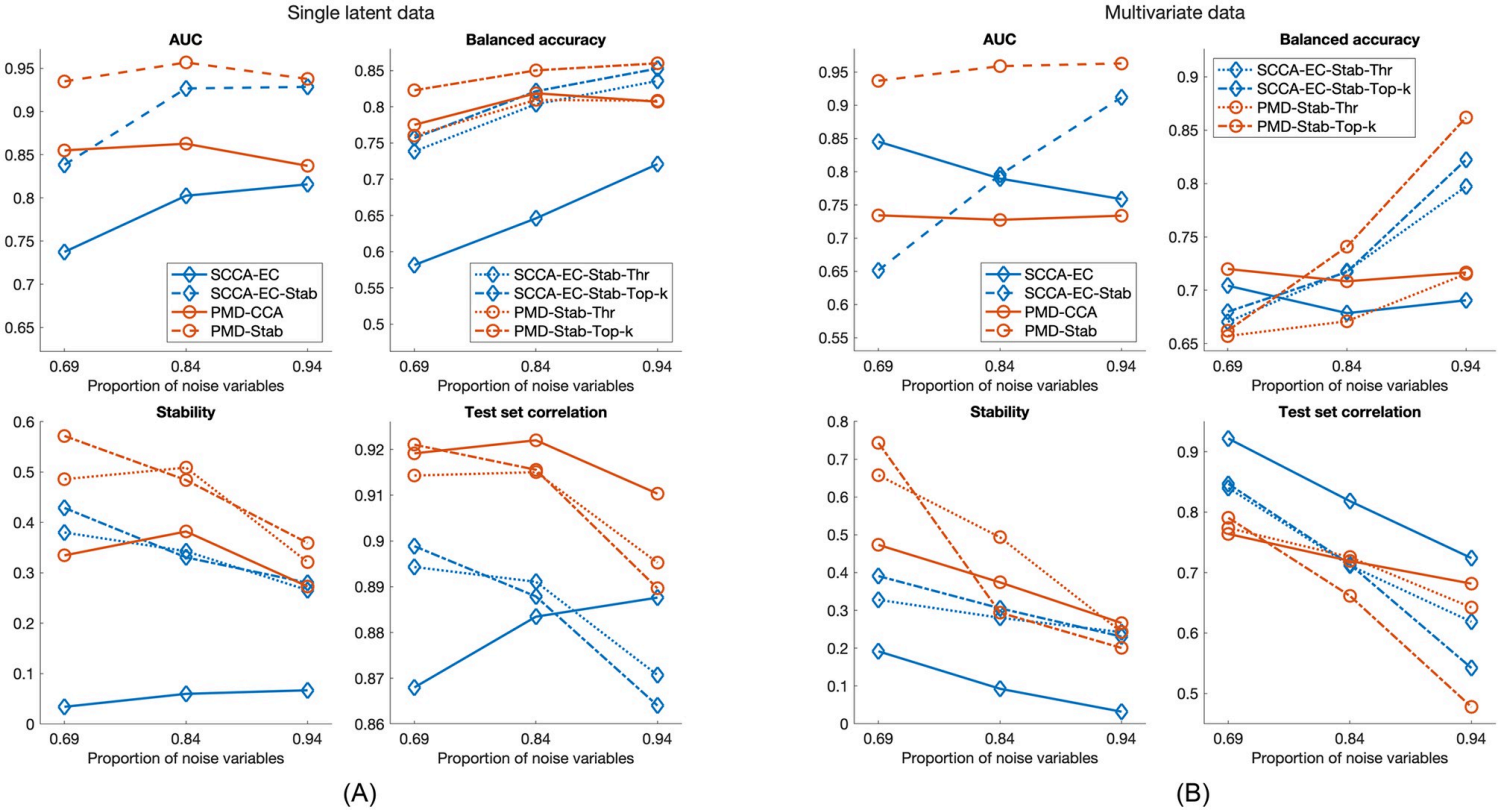
Results for simulated data sets. The base procedures are plotted with solid lines, while the dashed lines represent their different StabilityCCA extensions.

The results for single latent data are shown in Fig 2A. For SCCA-EC, we observe a substantial improvement from StabilityCCA across all three variable selection metrics in all scenarios with not much difference between the Top-k and Thr models. For test set correlation there is little difference with the base procedure performing slightly better in the sparsest case.

Similar, if not as pronounced improvements are found for PMD-CCA. Notably, PMD-CCA alone clearly outperforms SCCA-EC, and even its StabilityCCA extension in some cases. All in all, we observe clear improvements from StabilityCCA in variable selection, in particular stability, with not much difference in model performance, independent of the SCCA method used.

The multivariate data results, shown in Fig 2B, are more mixed. For SCCA-EC, while stability is still improved, AUC and BA are in some cases higher for the base procedure alone. It appears that SCCA-EC benefits from stability selection more in sparser scenarios. Test set correlation on the other hand is always higher for the base procedure model.

For PMD-CCA, there is still a clear improvement in AUC. The Top-k and Thr selections diverge somewhat: the Top-k model has much better BA in the sparse cases, and is the most stable in the densest case while dropping even below the base procedure for sparser cases. The Thr model has more even stability performance and outperforms Top-k for test set correlation. Interestingly, the results also differ from the single latent data in that SCCA-EC now outperforms PMD-CCA for AUC and test set correlation.

In summary, in our simulated data, adding the stability selection framework to SCCA seems to improve variable selection and in particular selection stability with not much difference in model performance. It appears that PMD-CCA is more stable than SCCA-EC to begin with, and does not benefit as much from StabilityCCA.

### Real data

We further evaluated StabilityCCA on two different omics combinations from an IBD data set. IBD is a group of gastrointestinal conditions, the most common forms being Crohn’s disease (CD) and ulcerative colitis (UC). The data comprises fecal metagenomics and metabolomics of individuals with CD (88), UC (76) and healthy controls (56), and was published in [23]. There are two alternative feature sets for metagenomics: species (201 variables) or enzymes (2113 variables), and 466 metabolite variables.

We downloaded abundance tables from the electronic supplementary materials to [23]. All data was transformed using the generalised log transformation: $\text{glog}\left(x\right)=\text{log}\left(x+\sqrt{x^{2}+10^{-8}}\right)$ [24, 25]. Variables which had zero variance were removed. Finally, each column (variable) was normalised to have mean zero and variance one.

Since here we obviously do not have a ground truth to compare against, only stability and model performance were evaluated. Non-overlapping training and test sets of size 100 were randomly drawn 25 times. Stability was measured across the selections in these 25 sets and test set correlation is reported as the average over them as before. Hyperparameter tuning was the same as in the simulation experiments.

**Fig 3:**
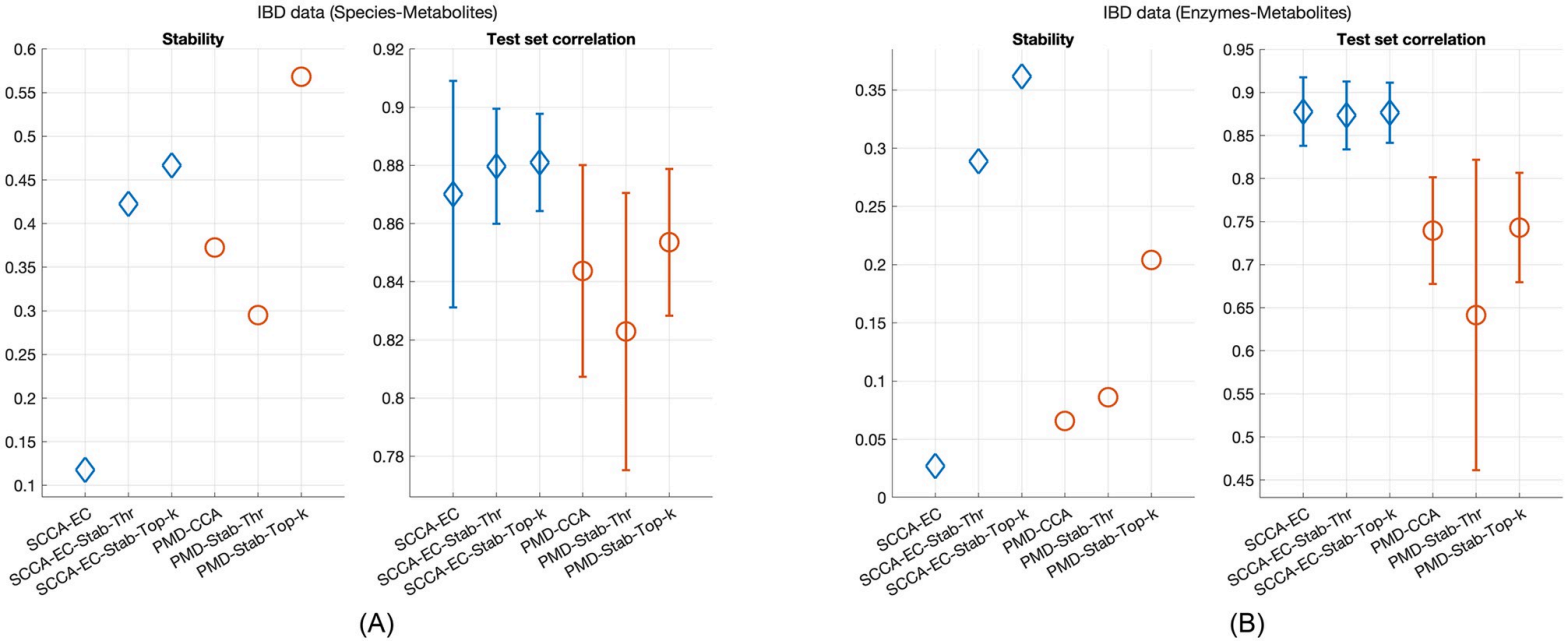
Results for real data. The whiskers display one standard deviation for the test set correlations.

The results for the species-metabolites case are shown in Fig 3A. For SCCA-EC, there is again a very notable improvement in stability from StabilityCCA, with the Top-k model being slightly more stable than Thr. There is little change in model performance. For PMD-CCA, the base procedure is now again much more stable than SCCA-EC. The Thr-extension underperforms, having worse stability than the base procedure alone while PMD-CCA-Top-k has the highest stability of all methods. However, for model performance, SCCA-EC outperforms across the board.

In the enzymes-metabolites case in Fig 3B, SCCA-EC performance is very similar to the previous case. For PMD-CCA, the base procedure selection is no longer stable. While stability is again improved by the Top-k selection, both SCCA-EC extensions are now more stable. In terms of model performance, SCCA-EC is now clearly better for both the base procedure and the StabilityCCA extensions.

All in all, our experiments on real data show that StabilityCCA greatly improves variable selection stability with no difference or even small improvements in model performance. The Top-k model appears to be the better selection strategy. The results also suggest that of the two SCCA methods tested, SCCA-EC might be better suited for these types of multi-omics data.

### Canonical correlations are linked to disease status

We further illustrate the use of StabilityCCA by taking a closer look at the IBD data set. Based on the results presented above, we choose SCCA-EC as the base procedure and Top-k as the variable selection method. However, as mentioned before, in practice we would likely not wish to attempt to optimise *k* over all possible values but rather choose a relatively low value based on practical considerations such as ease of interpretability.

**Fig 4:**
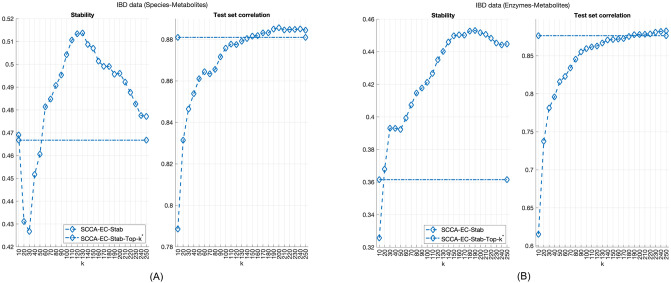
Top-k model performance for different *k* values in the IBD data set. The constant lines correspond to the average performance when an optimal value k* was selected through hyperparameter tuning.

**Fig 5:**
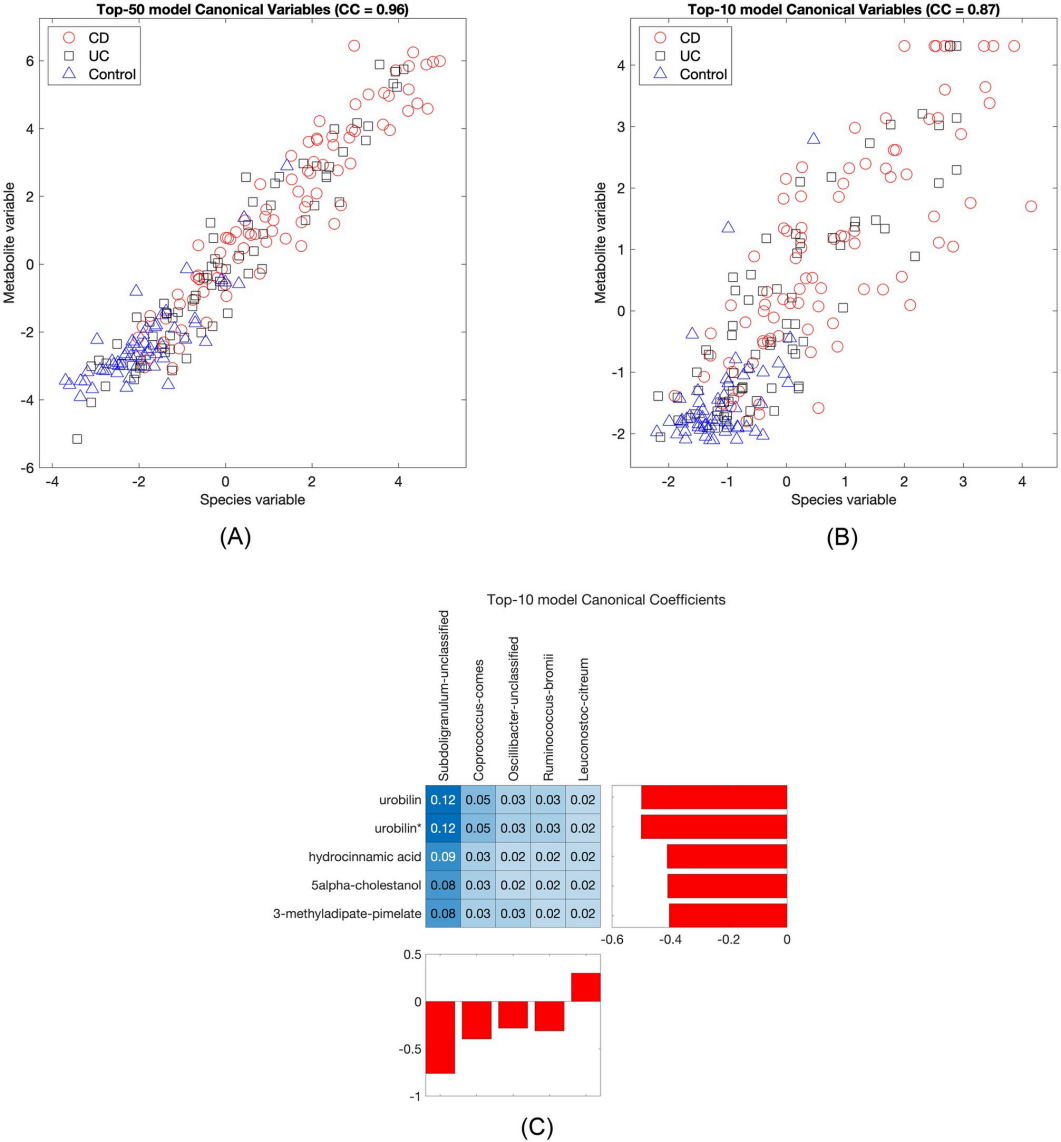
StabilityCCA models for species-metabolites IBD data. (A) Top-50 model canonical variables. The canonical correlation (CC) is shown above the plot. (B) Top-10 model canonical variables. (C) Canonical coefficients and pairwise contributions to the canonical correlation for the top-10 model. (*) match to a standard with isomeric forms that could not be differentiated.

Indeed, when we investigated how increasing *k* will affect model performance, we found that while the average test set correlation does keep increasing up to *k* = 150, very little is gained beyond *k* = 50, and most of the canonical correlation is already captured with just ten variables (see Fig 4A). Moreover, as we see in Fig 5A and 5B, the top-50 and top-10 models show qualitatively the same behaviour.

The plots show the canonical variables for the two views, species-composition of the metagenome and metabolomics. Each point corresponds to a sample and the IBD status of the individual has been highlighted. We see that there is a clustering happening, separating IBD samples from controls. To quantify this pattern, we calculated the receiver operating characteristic curves for disease status using the sum of the canonical variables, **X** **a** + **Y** **a**, as the score: the AUC was 0.88 (IBD v Control, 0.94 for CD v Control and 0.81 for UC v Control) for the top-10 model and 0.86 for the top-50 model (0.93 for CD v Control and 0.79 for UC v Control). This suggests several things: the uncovered connections between the metagenome and the metabolome are strongly linked to disease status in the IBD data, and while this connection is potentially very high-dimensional, its core can be captured by the top variables highlighted by StabilityCCA.

Table 1 shows the top-10 variables and their stability scores. The highest ranked variable, species *Subdoligranulum* unspecified, has a score close to one, indicating that it is almost always selected. Another prominent variable is the metabolite urobilin. Fig 5C displays the canonical coefficients and the variables’ pairwise contributions: the contribution from variable *j* of the **X**-view (species) and variable *k* from the **Y**-view (metabolites) is

$$\begin{array}{c} \frac{a_{j}X_{j}^{T}Y_{k}b_{k}}{\left\langle Xa,Yb\right\rangle } \end{array}$$ (9)

and it is the proportion of the total canonical correlation (Eq (1)) due to the correlation between *j* and *k*. Accordingly, the highest contributions come from *Subdoligranulum* unspecified, and highest pairwise contributions are between *Subdoligranulum* and urobilin.

**Table 1:** Top-10 variables from the species-metabolites data set.

Name	Type	Stability score
Subdoligranulum unclassified	Species	0.95
Urobilin	Metabolite	0.86
Hydrocinnamic acid	Metabolite	0.71
Coprococcus comes	Species	0.65
Ruminococcus bromii	Species	0.63
Urobilin*	Metabolite	0.63
5alpha-cholestanol	Metabolite	0.63
Oscillibacter unclassified	Species	0.62
3-methyladipate-pimelate	Metabolite	0.58
Leuconostoc citreum	Species	0.58

(*) match to a standard with isomeric forms that could not be differentiated.

Many of the top-10 variables have already been previously connected to IBD. More specifically, previous studies have shown a connection between IBD and decreases in the abundance or levels of *Subdoligranulum* species [26–29], urobilin [30, 31], hydrocinnamic acid [31], the genus *Coprococcus* [32–36], *Ruminococcus bromii* [27, 37], and *Oscillibacter* [36, 38].

**Fig 6:**
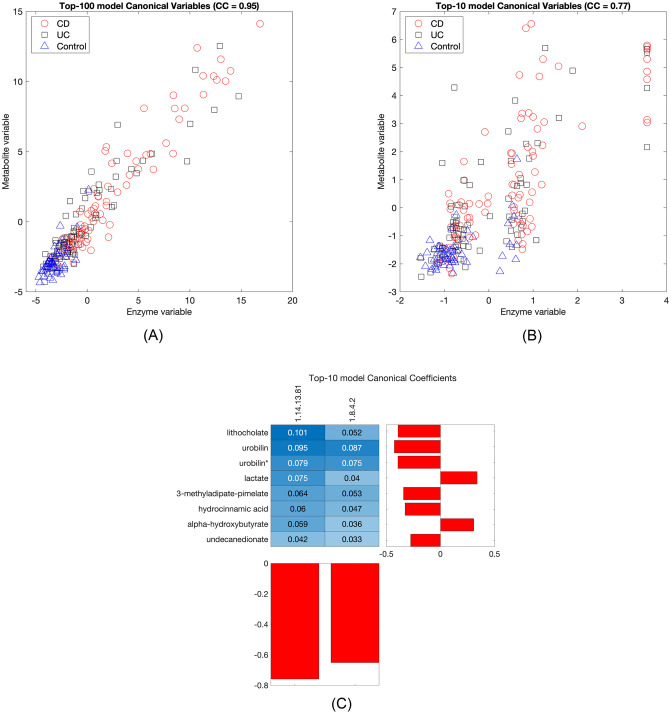
StabilityCCA models for enzymes-metabolites IBD data. (A) Top-100 model canonical variables. The canonical correlation (CC) is shown above the plot. (A) Top-10 model canonical variables. (C) Canonical coefficients and pairwise contributions to the canonical correlation for the top-10 model. (*) match to a standard with isomeric forms that could not be differentiated.

A similar pattern is found when the enzymes feature set is used as the metagenomics view. Fig 6 shows the canonical variables for a top-100 and a top-10 model. Again using the sum of the variables as a score, the AUC is 0.90 (IBD v Control, 0.95 for CD v Control and 0.84 for UC v Control) for the top-100 model and 0.88 for the top-10 model (0.94 for CD v Control and 0.81 for UC v Control), showing that while in this case more top variables are needed to achieve the highest test correlations (see Fig 4B), the underlying biological pattern is already captured by just ten.

Table 2 shows the top-10 variables and their stability scores. Many of the same metabolites are present. Of the new ones, lithocholate potentially inhibits inflammation [39, 40] and alpha-hydroxybutyrate has been found to be elevated in IBD [41, 42]. The canonical coefficients and pairwise contributions are shown in Fig 6C. The highest contribution is due to lithocholate and the enzyme 1.8.4.2: Protein-disulfide reductase (glutathione)—the highest ranked variable and the highest ranked enzyme variable.

**Table 2:** Top-10 variables from the enzymes-metabolites data set.

Name	Type	Stability score
Lithocholate	Metabolite	0.87
Undecanedionate	Metabolite	0.71
Hydrocinnamic acid	Metabolite	0.69
Urobilin	Metabolite	0.69
3-methyladipate-pimelate	Metabolite	0.66
1.14.13.81: Magnesium-protoporphyrin IX monomethyl ester (oxidative) cyclase	Enzyme	0.65
Urobilin*	Metabolite	0.64
Alpha-hydroxybutyrate	Metabolite	0.63
Lactate	Metabolite	0.62
1.8.4.2: Protein-disulfide reductase (glutathione)	Enzyme	0.62

(*) match to a standard with isomeric forms that could not be differentiated.

**Fig 7:**
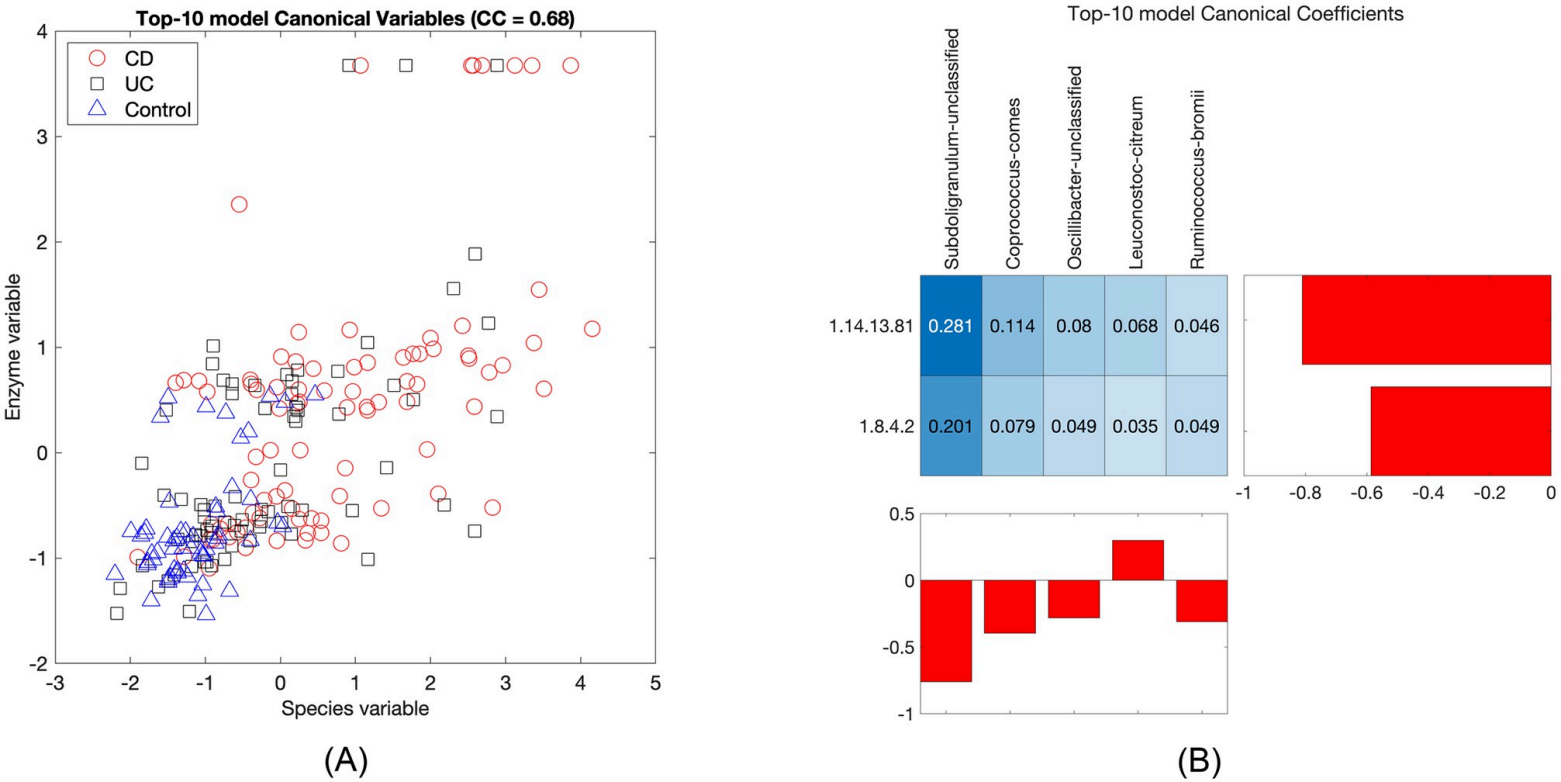
Canonical variables (A) and coefficients (B) for the species and enzymes views, taken from the top-10 species-metabolites model and the top-10 enzymes-metabolites model.

Given that the same patterns were observed with both the species and the enzymes representation of the metagenome, it would be interesting to relate the two to each other—after all, they are alternative depictions of the same underlying system. We can do this directly by taking the canonical coefficients from the species-metabolites model and the enzymes-metabolites model. The result is shown in Fig 7. The resulting canonical correlation is 0.68. The highest pairwise contribution is between *Subdoligranulum* and the protein-disulfide reductase. Both in turn had high interactions with the same metabolites, most notably the two features matched to urobilin.

## Discussion

We have presented a novel approach to variable selection in multi-view data: StabilityCCA. The method combines stability selection with sparse CCA to assess variable importance and select stable sets of variables. We tested the method with two different sparse CCA approaches, using both simulated and real data. The results show that by applying the stability selection framework, we can improve the ability to select true variables as well as the stability of the selection, regardless of the sparse CCA approach used. Importantly, in real multi-omics data, stability of selected feature sets was greatly improved by StabilityCCA.

We applied StabilityCCA to an IBD gut microbiome data set. Suggested causes for IBD include interactions between genetic and environmental factors [43]. In particular, the microbiome has been heavily implicated, with changes in microbiota composition and diversity being routinely observed. However, the exact causative mechanisms remain unknown [44, 45]. Since the metabolome acts as the interface mediating host and microbiota interaction, a metagenome-metabolome multi-omics analysis is a promising avenue to a better understanding of IBD pathology.

We found the canonical correlations between the gut metagenome and metabolome to be strongly linked to the disease. The connection was stronger for Crohn’s disease than for ulcerative colitis, in line with previous analysis of the same data [23]. With StabilityCCA, we were able to pinpoint a relatively small set of variables most responsible for this observed structure, many of which have already been linked to IBD.

While the exact aetiology of IBD remains unclear, the role of butyrate and other short-chain fatty acids (SCFAs) in reducing inflammation and maintaining the integrity of the epithelium barrier has been extensively investigated [46]. Accordingly, at least two of the microbial species highlighted by our analysis, *Subdoligranulum* unspecified and *Ruminococcus bromii*, have been identified as SCFA producers [26, 37]. The hypothesis is that a reduced abundance of these beneficial bacteria lead to decreased levels of SCFAs, which in turn lead to inflammation and epithelium breakdown.

In a similar vein, lithocholate, the top variable in the ezymes-metabolites scenario, has been proposed to inhibit epithelial apoptosis and promote barrier function [39, 40]. Another metabolite within the enzymes-metabolites top-10, alpha-hydroxybutyrate, is a potential biomarker for autoimmune disease [42]. Futhermore, it is part of propanoate metabolism: changes in propanoate and butanoate metabolism within the microbiome have been linked to IBD [45].

There are some important limitations to our analysis. As an unsupervised method, CCA, and by extension StabilityCCA, may not optimally fit settings that require supervised prediction. Also, the canonical correlations cannot be directly interpreted as causal dependencies, and hence further study is needed to provide a full picture of the underlying biological significance of the findings.

Additionally, we have focussed here on extending stability selection to CCA which is designed for two views only. There are several alternative ways to extend CCA to three or more views [47, 48], and further research is needed to understand whether our approach could be used with these methods as well.

The fact that the connection between the microbiome and IBD arises “serendipitously” from the data—meaning that disease status was not part of the analysis—arguably lends further credence both to the hypothesis that IBD is connected to the microbiota and the theory that this influence is mediated by the metabolome. By revealing a joint structure of the metagenome and the metabolome, the model points to a good starting point for future study which will hopefully shine more light on the exact mechanisms behind IBD, demonstrating the promise in multi-view analysis.

## References

[1] Hasin Y, Seldin M, Lusis A. Multi-omics approaches to disease. Genome biology. 2017;18(1):1–15. doi: 10.1186/s13059-017-1215-1 28476144 PMC5418815

[2] Pinu FR, Beale DJ, Paten AM, Kouremenos K, Swarup S, Schirra HJ, et al. Systems biology and multi-omics integration: viewpoints from the metabolomics research community. Metabolites. 2019;9(4):76. doi: 10.3390/metabo9040076 31003499 PMC6523452

[3] Nguyen ND, Wang D. Multiview learning for understanding functional multiomics. PLoS computational biology. 2020;16(4):e1007677. doi: 10.1371/journal.pcbi.1007677 32240163 PMC7117667

[4] Reel PS, Reel S, Pearson E, Trucco E, Jefferson E. Using machine learning approaches for multi-omics data analysis: A review. Biotechnology Advances. 2021;49:107739. doi: 10.1016/j.biotechadv.2021.107739 33794304

[5] Picard M, Scott-Boyer MP, Bodein A, Périn O, Droit A. Integration strategies of multi-omics data for machine learning analysis. Computational and Structural Biotechnology Journal. 2021;19:3735–3746. doi: 10.1016/j.csbj.2021.06.030 34285775 PMC8258788

[6] Chong J, Xia J. Computational approaches for integrative analysis of the metabolome and microbiome. Metabolites. 2017;7(4):62. doi: 10.3390/metabo7040062 29156542 PMC5746742

[7] Krassowski M, Das V, Sahu SK, Misra BB. State of the field in multi-omics research: From computational needs to data mining and sharing. Frontiers in Genetics. 2020;11:610798. doi: 10.3389/fgene.2020.610798 33362867 PMC7758509

[8] Bersanelli M, Mosca E, Remondini D, Giampieri E, Sala C, Castellani G, et al. Methods for the integration of multi-omics data: mathematical aspects. BMC bioinformatics. 2016;17(2):167–177. doi: 10.1186/s12859-015-0857-9 26821531 PMC4959355

[9] Xu H, Caramanis C, Mannor S. Sparse algorithms are not stable: A no-free-lunch theorem. IEEE transactions on pattern analysis and machine intelligence. 2011;34(1):187–193. 21844627 10.1109/TPAMI.2011.177

[10] Bousquet O, Elisseeff A. Stability and generalization. The Journal of Machine Learning Research. 2002;2:499–526.

[11] Zhao J, Xie X, Xu X, Sun S. Multi-view learning overview: Recent progress and new challenges. Information Fusion. 2017;38:43–54. doi: 10.1016/j.inffus.2017.02.007

[12] Meinshausen N, Bühlmann P. Stability selection. Journal of the Royal Statistical Society: Series B (Statistical Methodology). 2010;72(4):417–473. doi: 10.1111/j.1467-9868.2010.00740.x

[13] Shah RD, Samworth RJ. Variable selection with error control: another look at stability selection. Journal of the Royal Statistical Society: Series B (Statistical Methodology). 2013;75(1):55–80. doi: 10.1111/j.1467-9868.2011.01034.x

[14] Hotelling H. Relations between two sets of variates. In: Breakthroughs in statistics. Springer; 1992. p. 162–190.

[15] Uurtio V, Monteiro JM, Kandola J, Shawe-Taylor J, Fernandez-Reyes D, Rousu J. A tutorial on canonical correlation methods. ACM Computing Surveys (CSUR). 2017;50(6):1–33. doi: 10.1145/3136624

[16] Parkhomenko E, Tritchler D, Beyene J. Genome-wide sparse canonical correlation of gene expression with genotypes. In: BMC proceedings. vol. 1. Springer; 2007. p. 1–5. 10.1186/1753-6561-1-s1-s119 PMC2367499 18466460

[17] González I, Déjean S, Martin PG, Baccini A. CCA: An R package to extend canonical correlation analysis. Journal of Statistical Software. 2008;23(12):1–14.

[18] Witten DM, Tibshirani R, Hastie T. A penalized matrix decomposition, with applications to sparse principal components and canonical correlation analysis. Biostatistics. 2009;10(3):515–534. doi: 10.1093/biostatistics/kxp008 19377034 PMC2697346

[19] Uurtio V, Bhadra S, Rousu J. Large-scale sparse kernel canonical correlation analysis. In: International Conference on Machine Learning. PMLR; 2019. p. 6383–6391.

[20] Parkhomenko E, Tritchler D, Beyene J. Sparse canonical correlation analysis with application to genomic data integration. Statistical applications in genetics and molecular biology. 2009;8(1). doi: 10.2202/1544-6115.1406 19222376

[21] Rodosthenous T, Shahrezaei V, Evangelou M. Integrating multi-OMICS data through sparse canonical correlation analysis for the prediction of complex traits: a comparison study. Bioinformatics. 2020;36(17):4616–4625. doi: 10.1093/bioinformatics/btaa530 32437529 PMC7750936

[22] Nogueira S, Sechidis K, Brown G. On the Stability of Feature Selection Algorithms. Journal of Machine Learning Research. 2018;18(174):1–54.

[23] Franzosa EA, Sirota-Madi A, Avila-Pacheco J, Fornelos N, Haiser HJ, Reinker S, et al. Gut microbiome structure and metabolic activity in inflammatory bowel disease. Nature microbiology. 2019;4(2):293–305. doi: 10.1038/s41564-018-0306-4 30531976 PMC6342642

[24] Durbin BP, Hardin JS, Hawkins DM, Rocke DM. A variance-stabilizing transformation for gene-expression microarray data. Bioinformatics. 2002;18(suppl_1):S105–S110. doi: 10.1093/bioinformatics/18.suppl_1.S105 12169537

[25] Huber W, Von Heydebreck A, Sültmann H, Poustka A, Vingron M. Variance stabilization applied to microarray data calibration and to the quantification of differential expression. Bioinformatics. 2002;18(suppl_1):S96–S104. doi: 10.1093/bioinformatics/18.suppl_1.S96 12169536

[26] Xia Y, Wang J, Fang X, Dou T, Han L, Yang C. Combined analysis of metagenomic data revealed consistent changes of gut microbiome structure and function in inflammatory bowel disease. Journal of Applied Microbiology. 2021;131(6):3018–3031. doi: 10.1111/jam.15154 34008889

[27] Mondot S, Kang S, Furet JP, Aguirre de Cárcer D, McSweeney C, Morrison M, et al. Highlighting new phylogenetic specificities of Crohn’s disease microbiota. Inflammatory bowel diseases. 2011;17(1):185–192. doi: 10.1002/ibd.21436 20722058

[28] Chen D, Li Y, Sun H, Xiao M, Lv N, Liang S, et al. P854 Insights into alteration of gut microbiota in inflammatory bowel disease patients with and without Clostridium difficile infection. Journal of Crohn’s and Colitis. 2019;13(Supplement_1):S551–S552. doi: 10.1093/ecco-jcc/jjy222.978

[29] Pisani A, Rausch P, Ellul S, Bang C, Tabone T, Marantidis Cordina C, et al. P685 Gut microbiota in patients with Inflammatory Bowel Disease during remission. Journal of Crohn’s and Colitis. 2021;15(Supplement_1):S604–S605. doi: 10.1093/ecco-jcc/jjab076.805

[30] Lloyd-Price J, Arze C, Ananthakrishnan AN, Schirmer M, Avila-Pacheco J, Poon TW, et al. Multi-omics of the gut microbial ecosystem in inflammatory bowel diseases. Nature. 2019;569(7758):655–662. doi: 10.1038/s41586-019-1237-9 31142855 PMC6650278

[31] Santoru ML, Piras C, Murgia A, Palmas V, Camboni T, Liggi S, et al. Cross sectional evaluation of the gut-microbiome metabolome axis in an Italian cohort of IBD patients. Scientific reports. 2017;7(1):1–14. doi: 10.1038/s41598-017-10034-5 28842640 PMC5573342

[32] Turpin W, Goethel A, Bedrani L, Croitoru K MDCM. Determinants of IBD heritability: genes, bugs, and more. Inflammatory bowel diseases. 2018;24(6):1133–1148. doi: 10.1093/ibd/izy085 29701818 PMC6093195

[33] Kong L, Lloyd-Price J, Vatanen T, Seksik P, Beaugerie L, Simon T, et al. Linking strain engraftment in fecal microbiota transplantation with maintenance of remission in Crohn’s disease. Gastroenterology. 2020;159(6):2193–2202. doi: 10.1053/j.gastro.2020.08.045 32860788 PMC7725862

[34] Nishino K, Nishida A, Inoue R, Kawada Y, Ohno M, Sakai S, et al. Analysis of endoscopic brush samples identified mucosa-associated dysbiosis in inflammatory bowel disease. Journal of gastroenterology. 2018;53(1):95–106. doi: 10.1007/s00535-017-1384-4 28852861

[35] Shaw KA, Bertha M, Hofmekler T, Chopra P, Vatanen T, Srivatsa A, et al. Dysbiosis, inflammation, and response to treatment: a longitudinal study of pediatric subjects with newly diagnosed inflammatory bowel disease. Genome medicine. 2016;8(1):1–13. doi: 10.1186/s13073-016-0331-y 27412252 PMC4944441

[36] Pisani A, Rausch P, Bang C, Ellul S, Tabone T, Marantidis Cordina C, et al. Dysbiosis in the Gut Microbiota in Patients with Inflammatory Bowel Disease during Remission. Microbiology Spectrum. 2022; p. e00616–22. doi: 10.1128/spectrum.00616-22 35532243 PMC9241752

[37] Rajilić-Stojanović M, Shanahan F, Guarner F, de Vos WM. Phylogenetic analysis of dysbiosis in ulcerative colitis during remission. Inflammatory bowel diseases. 2013;19(3):481–488. doi: 10.1097/MIB.0b013e31827fec6d 23385241

[38] Papa E, Docktor M, Smillie C, Weber S, Preheim SP, Gevers D, et al. Non-invasive mapping of the gastrointestinal microbiota identifies children with inflammatory bowel disease. PloS one. 2012;7(6):e39242. doi: 10.1371/journal.pone.0039242 22768065 PMC3387146

[39] Ward JB, Lajczak NK, Kelly OB, O’Dwyer AM, Giddam AK, Ní Gabhann J, et al. Ursodeoxycholic acid and lithocholic acid exert anti-inflammatory actions in the colon. American Journal of Physiology-Gastrointestinal and Liver Physiology. 2017;312(6):G550–G558. doi: 10.1152/ajpgi.00256.2016 28360029

[40] Lajczak-McGinley NK, Porru E, Fallon CM, Smyth J, Curley C, McCarron PA, et al. The secondary bile acids, ursodeoxycholic acid and lithocholic acid, protect against intestinal inflammation by inhibition of epithelial apoptosis. Physiological reports. 2020;8(12):e14456. doi: 10.14814/phy2.14456 32562381 PMC7305237

[41] Santoru ML, Piras C, Murgia F, Leoni VP, Spada M, Murgia A, et al. Metabolic Alteration in Plasma and Biopsies from Patients with IBD. Inflammatory Bowel Diseases. 2021;27(8):1335–1345. doi: 10.1093/ibd/izab012 33512485

[42] Tsoukalas D, Fragoulakis V, Papakonstantinou E, Antonaki M, Vozikis A, Tsatsakis A, et al. Prediction of autoimmune diseases by targeted metabolomic assay of urinary organic acids. Metabolites. 2020;10(12):502. doi: 10.3390/metabo10120502 33302528 PMC7764183

[43] Glassner KL, Abraham BP, Quigley EM. The microbiome and inflammatory bowel disease. Journal of Allergy and Clinical Immunology. 2020;145(1):16–27. doi: 10.1016/j.jaci.2019.11.003 31910984

[44] Nagalingam NA, Lynch SV. Role of the microbiota in inflammatory bowel diseases. Inflammatory bowel diseases. 2012;18(5):968–984. doi: 10.1002/ibd.21866 21936031

[45] Kostic AD, Xavier RJ, Gevers D. The microbiome in inflammatory bowel disease: current status and the future ahead. Gastroenterology. 2014;146(6):1489–1499. doi: 10.1053/j.gastro.2014.02.009 24560869 PMC4034132

[46] Zhuang X, Li T, Li M, Huang S, Qiu Y, Feng R, et al. Systematic review and meta-analysis: short-chain fatty acid characterization in patients with inflammatory bowel disease. Inflammatory bowel diseases. 2019;25(11):1751–1763. doi: 10.1093/ibd/izz188 31498864

[47] Kettenring JR. Canonical analysis of several sets of variables. Biometrika. 1971;58(3):433–451. doi: 10.1093/biomet/58.3.433

[48] Luo Y, Tao D, Ramamohanarao K, Xu C, Wen Y. Tensor canonical correlation analysis for multi-view dimension reduction. IEEE transactions on Knowledge and Data Engineering. 2015;27(11):3111–3124. doi: 10.1109/TKDE.2015.2445757

